# Media Messages and Perception of Risk for Ebola Virus Infection, United States

**DOI:** 10.3201/eid2301.160589

**Published:** 2017-01

**Authors:** Tara Kirk Sell, Crystal Boddie, Emma E. McGinty, Keshia Pollack, Katherine Clegg Smith, Thomas A. Burke, Lainie Rutkow

**Affiliations:** UPMC Center for Health Security, Baltimore, Maryland, USA (T.K. Sell, C. Boddie);; Johns Hopkins Bloomberg School of Public Health, Baltimore (T.K. Sell, C. Boddie, E.E. McGinty, K. Pollack, K.C. Smith, L. Rutkow);; United States Environmental Protection Agency, Washington, DC, USA (T.A. Burke)

**Keywords:** communicable diseases, decision making, disease outbreaks, hemorrhagic fever, Ebola, Ebola virus infection, Ebola virus disease, mass media, risk, viruses, United States

## Abstract

News media have been blamed for sensationalizing Ebola in the United States, causing unnecessary alarm. To investigate this issue, we analyzed US-focused news stories about Ebola virus disease during July 1–November 30, 2014. We found frequent use of risk-elevating messages, which may have contributed to increased public concern.

The 2014–15 outbreak of Ebola virus disease (EVD) generated much news media coverage and highlighted the role of news media with regard to providing information about risks to the public (*1**–**3*). Research shows that the news media can influence knowledge and perceptions about a topic (*4**–**6*)*.* The way risks are discussed and communicated (often through news coverage) can also affect how risk is perceived (*7**–**9*). Our objective was to analyze the volume and content of messages promoted in US news media with regard to risk for EVD and to examine how these messages relate to risk-perception theory.

## The Study

Using established methods, we analyzed EVD coverage from 12 news sources (9 print, 3 television) published July 1–November 30, 2014 (Technical Appendix Table 1). News media stories were collected through searches of LexisNexis, ProQuest, and NewsBank online archives by using the term “Ebola.” The search yielded 2,989 news stories, which were reviewed to determine if they met inclusion criteria (focus on US-associated EVD). The 374 stories that did not place EVD in a US context were included in our analysis of news volume only. The final sample for content analysis included 1,262 news stories and opinion pieces from print and television sources.

Our coding instrument contained 9 risk-elevating messages with characteristics that could increase perception of risk and 5 risk-minimizing messages with characteristics that could decrease perception of risk (Technical Appendix Tables 2–4), developed according to the risk perception framework of Slovic (*7*). To assess inter-rater reliability, we coded a random sample of 15% of news stories. Most items met conventional standards for adequate reliability; κ values were >0.69 (*10*). For 4 items, κ values were slightly below this threshold but raw percentage agreement was high (90%–94%); therefore, these items were also included (Technical Appendix Table 3). We assessed news story content about the EVD outbreak by calculating the proportion of stories that mentioned each EVD-associated message over the study period.

The volume of US-focused news coverage of the EVD outbreak peaked slightly after the arrival (August 2, 2014) of the first patient transported to the United States for treatment and increased much more after a case was diagnosed in Dallas, Texas, USA, on September 30, 2014 (Figure). Overall, 96% of print and television news stories that covered EVD in the context of the United States included >1 risk-elevating messages, 55% of stories contained >1 risk-minimizing messages, and 53% contained both message types. The most common risk-elevating messages (72%) concerned foreigners or travelers bringing Ebola virus to the United States. The most frequent risk-minimizing messages (32%) described scientific knowledge about EVD (Table).

**Figure:**
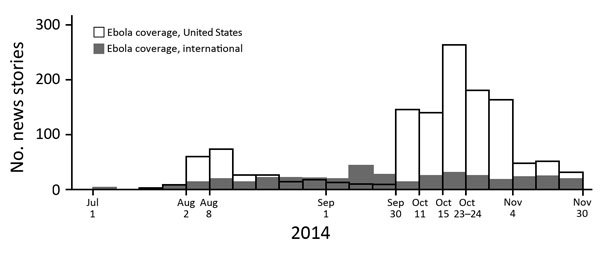
News coverage of Ebola virus disease comparing stories about Ebola in the United States and internationally, July–November 2014. Aug 2, first Ebola virus disease patient arrives in the United States; Aug 8, World Health Organization declares public health emergency of international concern; Sep 30, first case diagnosed in Dallas, Texas, USA; Oct 11, first case in healthcare worker; Oct 15, second case in healthcare worker; Oct 23–24, case diagnosed in New York, USA, and nurse quarantined; Nov 4, US midterm elections.

**Table:** Risk-related news media messages about Ebola virus disease, July–November 2014*

Messages	News stories with message, %						
	Print and TV, n = 1,262†	Ebola case/local controversy, n = 655‡	National, no Ebola case/ controversy, n = 607§	Conservative, n = 302¶	Liberal, n = 595#	Print, n = 1,109**	TV, n = 153††
That could increase perception of risk							
Lack of/limited availability of countermeasures to stop Ebola	17	13	21 (p<0.001)	11	19 (p<0.01)	17	20
Ebola causes deaths	66	64	68	70	65	66	65
Potential US outbreak/persons in the United States contracting Ebola	35	33	36	35	33	34	41
Inability to stop transmission/outbreak in the United States	7	4	9 (p<0.01)	4	6	6	7
Growth of the Ebola epidemic	23	17	30 (p<0.001)	14	26 (p<0.001)	21	36 (p<0.001)
Science does not understand Ebola (e.g., previous knowledge about the disease was wrong or expert advice was incorrect)	8	8	8	7	9	7	13 (p<0.05)
Ebola’s potential use in terrorism or as a biologic weapon	1	1	1	1	1	1	1
Ebola has an incubation period	34	34	35	37	33	33	43 (p<0.05)
Foreigners or travelers bringing Ebola to the United States	72	71	74	72	70	71	79 (p<0.05)
That could decrease perception of risk							
Lower Ebola death rates in the United States	5	4	6	3	4	4	10 (p<0.001)
Ability to stop transmission/outbreak in the United States	20	16	24 (p<0.01)	24	17 (p<0.01)	18	30 (p<0.01)
Low risks related to Ebola (e.g., low risk of the disease coming to the United States, low risk of someone transmitting the disease, low risks of school children acquiring Ebola)	28	25	30	25	27	26	42 (p<0.001)
How to prevent spread of Ebola	12	12	13	12	10	11	20 (p<0.05)
Description of scientific knowledge about Ebola (e.g., transmission dynamics or other known aspects of the disease)	32	30	33	29	30	31	35

*Time frame selected to capture potential differences before and after key US Ebola events. χ^2^ tests were used to test differences in the proportion of news stories mentioning each Ebola-related message in compared news sources.  †Sources included in all news stories: Atlanta Journal Constitution, Chicago Tribune, CNN Situation Room, Fort Worth Star-Telegram, Fox Special Report, NBC Nightly News, New York Daily News, New York Times, Orange County Register, Portland Press Herald, USA Today, and Washington Post. ‡New sources with an Ebola case or controversy in the locality: Atlanta Journal Constitution, Fort Worth Star-Telegram, New York Daily News, New York Times, and Portland Press Herald. §Nationally produced new sources or those without an Ebola case or controversy in the locality: Chicago Tribune, CNN Situation Room, Fox Special Report, NBC Nightly News, Orange County Register, USA Today, and Washington Post. ¶Conservative news sources: Fort Worth Star-Telegram, Fox Special Report, and New York Daily News. #Liberal news sources: Chicago Tribune, New York Times, and Washington Post. **Print news sources: Atlanta Journal Constitution, Chicago Tribune, Fort Worth Star-Telegram, New York Daily News, New York Times, Orange County Register, Portland Press Herald, USA Today, and Washington Post. ††TV news sources: CNN Situation Room, Fox Special Report, and NBC Nightly News.

Our analysis of news volume suggested that diagnosis of the first EVD case in Dallas and subsequent cases diagnosed in the United States were influential time points in the escalation of EVD outbreak news coverage, although internationally, the outbreak had reached historic levels months earlier. As noted elsewhere (*1**,**11*), the volume of EVD news was largely reduced after the US midterm elections. This reduction may reflect inclusion of EVD as a campaign issue late in the election cycle or may reflect lack of newly diagnosed cases in the United States.

The high frequency of risk-elevating messages in news coverage may have contributed to increased public concern about EVD in the United States, which was greater than the situation warranted. Consumers of news media would have been exposed to risk-elevating messages more often than risk-minimizing messages, potentially increasing their perception of risk for EVD. Risk messages of both types were more frequently included in television news than in print news, potentially leading to differences in perceived EVD risk among consumers of different news types. Although many factors can alter a message’s effectiveness, frequency of exposure to risk-related messages can alter public perception and contribute to social amplification of risk; even when coverage is balanced, reassuring messages may be less able to counter messages that increase perception of risk (*6**,**9*)*.* However, several messages that were seen significantly more frequently in liberal news sources (defined in Table) may have been associated with increasing awareness of specific issues, such as medical countermeasure development efforts and large-scale growth of the EVD epidemic.

The news media have been blamed for sensationalizing the EVD outbreak in the United States and unnecessarily alarming the public (*3*). Although the volume of news coverage may have influenced public attention, the content of analyzed news stories does not necessarily suggest that news media were reporting news about EVD in a hyperbolic or irresponsible manner. Comparison of opposing messages, such as the ability to stop transmission or the outbreak in the United States, which was more frequently mentioned than the inability to do so, suggests that some concerns may have resulted from the nature of the risk itself, rather than irresponsible news media coverage. Additionally, messages that were most inflammatory (e.g., science not understanding the disease, inability to stop Ebola in the United States, terrorism/use of Ebola as a bioweapon) were mentioned less frequently than nearly all other messages analyzed.

Although the methods used in this study do not allow for causal inference between news media coverage and public polling about EVD, comparison with public polling may provide useful context. EVD news volume roughly reflected changing levels of concern about EVD (*1**,**12**,**13*). News media coverage could have increased public concern, or public concern could have increased news coverage of risks. Despite widespread coverage of EVD, poll respondents were often misinformed about how the disease was spread; 85% of respondents indicated that a person was likely to get EVD via a sneeze or cough from a symptomatic person, and 48% believed that transmission could occur before symptoms appeared (*14*)*.* In our analysis, only 32% of news stories included scientific knowledge such as how the disease is spread. More in-depth and frequent coverage of the scientific aspects (and disease contagion pathways in particular) of a public health threat may prevent these types of misperceptions.

Our results should be considered in light of several limitations. First, the sample did not include all news types (e.g., talk radio, social media, local television, blogs) or international news sources. Furthermore, κ statistics for 4 items in the coding instrument were slightly below conventional reliability standards; however, these messages were either very common or rare, which can result in lower κ agreement (*15*). These items were thus included because of high raw percentage agreement. Although the process used to create and evaluate the coding instrument should have accounted for risk-elevating or risk-minimizing messages used frequently in coverage of EVD, some risk-related messages may have been unintentionally omitted and the imbalanced number of messages may have influenced our analysis of the overall frequency of message types. Furthermore, trends in news coverage may have been influenced by competing issues in the news cycle. Last, this study does not provide direct measurement of exposure to or influence of messages. Examination of competing messages within news stories and comparison of news sources such as blogs or international sources may be promising areas for future research.

## Conclusions

The 2014–15 Ebola outbreak provides a useful case for studying emerging outbreaks and other public health emergencies. Certain risk messages about Ebola were used more frequently than others by US news media, which may have affected risk perception during the outbreak.

Technical Appendix News coverage of Ebola virus disease in the United States, July–November 2014. Descriptions of news media sources, messages potentially increasing or decreasing perception of risk, coding instrument and inter-rater agreement, examples of each type of message.
