# Supplementary material for: Media Messages and Perception of Risk for Ebola Virus Infection, United States
Source: Emerg Infect Dis. 2017 Jan;23(1):108–11. doi: 10.3201/eid2301.160589 (PMC5176223; doi:10.3201/eid2301.160589)
Supplement: Technical Appendix — News coverage of Ebola virus disease in the United States, July–November 2014. Descriptions of news media sources, messages potentially increasing or decreasing perception of risk, coding instrument and inter-rater agreement, examples of each type of message. [file 16-0589-Techapp-s1.pdf]

Article DOI: http://dx.doi.org/10.3201/eid2301.160589

Media Messages and Perception of Risk for Ebola Virus Infection, United States

Technical Appendix

Technical Appendix Table 1. News media sources

**Sources***	Regional or National Newspaper†	Liberal or Conservative Ideology‡	Ebola Case or Controversy in the Locality in Which the News Source is Based§
Print			
Atlanta Journal Constitution	South Region	–¶	Ebola Case
Chicago Tribune	Midwest Region	Liberal	–
Fort Worth Star Telegram	South Region	Conservative	Ebola Case
New York Daily News	Northeast Region	Conservative	Ebola Case
New York Times	National	Liberal	Ebola Case
Orange County Register	West Region	–	–
Portland Press Herald	Northeast Region	–	Ebola Controversy
USA Today	National	–	–
Washington Post	National	Liberal	–
**Television¶**			
CNN Situation Room	–	–	–
Fox Special Report	–	Conservative	–
NBC Nightly News	–	–	–

*Sources were chosen to provide variation in 4 categories: 1) geographic location (U.S. census region); 2) liberal or conservative ideology#,** 3) localities that experienced Ebola cases or controversies or not and 4) news source type (print, TV). We selected the highest circulation/viewership news sources available in LexisNexis, ProQuest, or Newsbank meeting these non-mutually exclusive criteria.††,‡‡

†Newspaper region was determined by location of news sources in U.S. census regions, with at least one source from each region. Newspapers with national distribution were considered "National."

‡Classification of conservative or liberal ideology was based on endorsement of Democratic or Republican candidates in the 2012 presidential election and viewer clustering around specific news sources according to data collected by Pew Research Center.#,** Television news sources were selected to provide a potential comparison of information presented to readers or viewers.§§

§Dashes indicate that this news source was not included in either category for this analysis.

¶Although television news sources were headquartered in cities that had experienced Ebola cases, they are nationally produced and focused and were therefore not classified as containing local coverage.

#Peters G, Woolley JT. 2012 General Election Editorial Endorsements by Major Newspapers. The American Presidency Project. Ed. Woolley JT and Peters G. Santa Barbara, CA: University of California. 1999–2012. Nov 5, 2012. http://www.presidency.ucsb.edu/data/2012_newspaper_endorsements.php. Accessed January 4, 2016.

**Pew Research Center. Political Polarization and Media Habits. October 20, 2014. http://www.journalism.org/2014/10/21/political-polarization-media-habits/pj_14-10-21_mediapolarization-00/. Accessed January 4, 2016.

††Alliance for audited Media. Top 25 Newspapers for March 2013. http://auditedmedia.com/news/research-and-data/top-25-us-newspapers-for-march-2013/. Accessed January 4, 2016.

‡‡Pew Research Center. State of the News Media 2015. April 29, 2015. http://www.journalism.org/2015/04/29/state-of-the-news-media-2015/. Accessed January 4, 2016.

§§Chaffee S, Frank S. How Americans Get Political Information: print versus broadcast news. Annals of the American Academy of Political and Social Science 546:48–58, 1996.

Technical Appendix Table 2. Messages potentially increasing or decreasing perception of risk*

Ebola-related message	Factors that increase risk perception (Slovic et al.)†
Lack of/limited availability of countermeasures	Disease is not controllable
Potential U.S. outbreak/people in the United States contracting Ebola	Disease is not controllable
Inability to stop transmission/outbreak in the United States	Disease is not controllable
Ebola causes deaths	Disease is fatal
Growth of the Ebola epidemic	Risk associated with disease is increasing
Science does not understand Ebola (e.g., previous knowledge about the disease was wrong or expert advice was incorrect)	Risk is unknown to science
Ebola's potential use in terrorism or as a biologic weapon	Catastrophic and dread characteristic
Ebola has an incubation period	Delayed effect after exposure to the disease
Foreigners or travelers bringing Ebola to the United States	Disease is exotic and unknown
**Ebola-related message**	**Factors that decrease risk perception (Slovic et al.)**
Lower Ebola death rates in the United States	Disease may not be fatal
Ability to stop transmission/outbreak in the United States	Disease is controllable
How to prevent spread of Ebola	Risk can be reduced
Description of scientific knowledge about the disease (e.g., transmission dynamics or other known aspects of the disease)	Risk is known to science, observable, and known to those exposed
Low risks related to Ebola (e.g., low risk of the disease coming to the United States, low risk of someone transmitting the disease, low risks of school children acquiring Ebola)	Indicate lowered risks associated with disease

*Following established methodology,‡ an initial coding instrument of messages relevant to risk perception was developed using an informal news media scan then reviewed by two outside infectious disease and public health experts to identify any further risk-related messages about Ebola that they may have observed in their professional roles. The instrument was piloted on 60 news stories from the study time period that appeared in two news sources not included in the study sample (Wall Street Journal, CBS Evening News) and further refined.

†Slovic P. Perception of risk. Science. 1987;236(4799):280–285.

‡McGinty EE, Wolfson JA, Sell, TK, Webster DW. Common Sense or Gun Control? Political Communication and News Media Framing of Firearm Sale Background Checks after Newtown J Health Polit Policy Law. 2016;41(1):3–40.

Technical Appendix Table 3. Coding instrument and inter-rater agreement

Basic Coding and Exclusions			
Domain	Coding Scheme		
Coder ID	1 – TKS<br>2 – CB		
Story unique ID			
Outlet	1 – USA Today<br>2 – New York Times<br>3 – Washington Post<br>4 – Orange County Register<br>5 – Atlanta Journal Constitution<br>6 – Fort Worth StarTelegram<br>7 – Portland Press Herald<br>8 – Chicago Tribune<br>9 – New York Daily News<br>10 – Huffington Post<br>11 – NBC Nightly News<br>12 – CNN Situation Room<br>13 – Fox Special Report		
Date	DD/MM		
Exclusion 1: Word count	1 – article <100 words<br>0 – article > = 100 words		
Exclusion 2:	1 – Correction<br>2 – Book review<br>3 – Letter to the editor<br>4 – Solely business/stock<br>5 – Obituaries<br>6 – Duplicate<br>7 – Index only<br>8 – Introduction/lead in only<br>9 – Calendar/event report<br>10 – Advice column<br>11 – Mentioned in passing<br>12 – Other (fill in)<br>0 – News story, health/lifestyle, metro, op-ed/editorial		
Exclusion 3:	1 – International focus/no coverage U.S. related Ebola issues<br>0 – Includes content about Ebola coming to/in the United States		
**Code Only for Included Stories**			
Item	Coding Scheme		
Story word count			
**Message**		**Raw Agreement (%)**	**Kappa Statistic**
Message about lack of/ limited availability of countermeasures	1 = yes, 0 = no	96	.84
Message about the disease causing deaths	1 = yes, 0 = no	94	.85
Message about lower death rates in the United States	1 = yes, 0 = no	98	.70
Message about a potential U.S. outbreak/people in the United States getting ebola	1 = yes, 0 = no	87	.73
Message about ability to stop transmission in the United States (positive)	1 = yes, 0 = no	93	.77
Message about ability to stop transmission in the United States (negative)	1 = yes, 0 = no	94	.63
Message about growth of the epidemic	1 = yes, 0 = no	89	.72
Message about low risks	1 = yes, 0 = no	90	.76
Message about science not understanding the disease	1 = yes, 0 = no	94	.67
Message about terrorism or potential use as a biologic weapon	1 = yes, 0 = no	100	.80
Message about how to prevent spread	1 = yes, 0 = no	90	.64
Message about incubation period	1 = yes, 0 = no	96	.90
Message describing science about the risks	1 = yes, 0 = no	93	.83
Message about foreigners, travelers or borders (exotic)	1 = yes, 0 = no	90	.67

Technical Appendix Table 4. Examples of each type of message

Message Types Potentially Increasing Perception of Risk	Example messages
Lack of/limited availability of countermeasures	"The maker of ZMapp has no more of the drug, which was made in small quantities because of its early stage of development." – USA Today, 9/18/14
Potential U.S. outbreak/people in the United States contracting Ebola	"The first diagnosed case of Ebola is sending chills through much of the United States tonight and despite official assurances, there are worries that patient zero in Dallas could be just the beginning." – Fox Special Report, 10/1/14<br>"We knew a second case could be a reality and we've been preparing for this possibility…"– New York Daily News, 10/13/14
Inability to stop transmission/outbreak in the United States	"Our public health system is not ready to deal with a challenge like Ebola if the situation takes a turn for the worst." – USA Today, 10/2/14<br><br>"…yet another day where there were more questions raised about whether the government really has a handle on this." – Fox Special Report, 10/16/14
Ebola causes deaths	"Almost 1,000 people have died of Ebola since March." –New York Times, 8/8/14<br><br>"…she was caring for Thomas Eric Duncan. He died Oct. 8 at Texas Health Presbyterian Hospital in Dallas." – Fort Worth Star-Telegram, 10/22/14
Growth of the Ebola epidemic	"The Ebola outbreak could last for years and spread to many more countries if it is not controlled quickly." – USA Today, 9/17/14
Science does not understand Ebola (e.g., previous knowledge about the disease was wrong or expert advice was incorrect)	"…The more this virus circulates in West Africa, Wolf, the greater chance it has of mutating...change in the virus to lead to possibly becoming…aerosol[ized]."<br><br>– CNN Situation Room, 10/17/14<br>"We don't actually know with any great precision how Ebola is transmitted." – Fox Special Report, 10/15/14<br><br>"We have to rethink the way we address Ebola infection control, because even a single infection is unacceptable," Thomas Frieden, director of the Centers for Disease Control and Prevention, said in a news conference." – Washington Post, 10/13/14
Ebola's potential use in terrorism or as a biologic weapon	"Are you worried that terrorist groups also try to use Ebola as a weapon against the United States? Have you heard anything on that essentially getting sympathetic infected people to specifically travel to the United States in the hopes of infecting others, is that a concern?" – Fox Special Report, 10/3/14
Ebola has an incubation period	"North Texas officially became Ebola-free on Friday when the last of 177 people being monitored because of their exposure moved out of the 21-day virus incubation period with no symptoms of sickness." – Fort Worth Star-Telegram, 11/7/14
Foreigners or travelers bringing Ebola to the United States	"Late this afternoon, Ashoka Mukpo boarded a plan in Monrovia, Liberia. After some nine hours in the air, he'll land in Nebraska and be taken to the Nebraska Medical Center." – NBC Nightly News, 10/5/14<br><br>"A man who flew from Liberia to Dallas in September was diagnosed with Ebola on Tuesday…" – Washington Post, 10/1/14
Message Types Potentially Decreasing Perception of Risk	
Lower Ebola death rates in the United States	"Two nurses who helped treat him, Nina Pham and Amber Vinson, later became ill and tested positive for Ebola. They received prompt, specialized treatment and survived…as serious as the threat was to Pham and Vinson, the numbers can be seen as encouraging." – Fort-Worth Star Telegram, 11/7/14
Ability to stop transmission/outbreak in the United States	"We do not anticipate this will spread in the U.S…" –New York Times, 7/29/14<br>"In the United States, we have a good health infrastructure and effective precautionary standards. Both greatly contribute to creating a formidable barrier against the spread of Ebola in the United States" – Orange County Register, 8/14/14
How to prevent spread of Ebola	"A key element is that no skin can be exposed by doctors, nurses or technicians taking care of a person infected with Ebola, which is transmitted through direct contact with bodily fluids and tissue but is not airborne." Chicago Tribune, 10/21/14
Description of scientific knowledge about the disease (e.g., transmission dynamics or other known aspects of the disease)	Ebola is spread only through direct contact with an infected person's bodily fluids. People are not contagious until the begin showing symptoms." – Atlanta Journal Constitution, 10/2/14
Low risks related to Ebola (e.g., low risk of the disease coming to the United States, low risk of someone transmitting the disease, low risks of school children acquiring Ebola)	"The Centers for Disease Control and Prevention said there was no significant risk to the United States from the outbreak." – New York Times, 8/1/14

Message Types Potentially Increasing Perception of Risk	Example messages
	“Carnival said the CDC had notified it that a passenger on the Carnival Magic was a lab supervisor at the hospital and deemed to be “very low risk.”” – Chicago Tribune, 10/18/14
	“Schools Chancellor Carmen Fariña and city Health Commissioner Mary Bassett fired off a letter to school principals, laying out possible Ebola warning signs but emphasizing that the risk to staff and students is “very small.” – New York Daily News, 10/17/14
